# Transfer of lens-specific transcripts to retinal RNA samples may underlie observed changes in crystallin-gene transcript levels after ischemia

**Published:** 2007-02-08

**Authors:** Willem Kamphuis, Frederike Dijk, Willem Kraan, Arthur A.B. Bergen

**Affiliations:** 1 Department of Molecular Ophthalmogenetics, Netherlands of Neuroscience-KNAW, Amsterdam, The Netherlands; 2 Department of Cellular Quality Control, Netherlands Institute for Neuroscience-KNAW, Amsterdam, The Netherlands

## Abstract

**Purpose:**

Retinal ischemia appears to lead to alterations in retinal transcript levels of a group of genes known to be abundantly expressed in the lens. Our purpose is to study whether these alterations are truly the result of retinal ischemia or whether they could be caused by contamination of the retinal tissue with trace amounts of lens tissue.

**Methods:**

Changes occurring in the retinal gene expression profile after induction of retinal ischemia were assessed by oligonucleotide microarrays and by real-time quantitative PCR.

**Results:**

Microarray analysis of the retinal gene expression profile after 5 or 60 min ischemia showed altered transcript levels for a group of genes with functions related to "structural constituent of eye lens" (23 genes, predominantly crystallins). Subsequent qPCR assays for this set of genes showed extremely high variations in transcript levels between individual animals of both control and ischemia-treated groups. However, the relative transcript levels, or expression profile, of these genes was constant in all samples. The transcript levels of these genes were on average 2624-times higher in tissue samples isolated from the superficial layers of the total lens. Moreover, all 23 genes had high expression levels in lens compared to retina as was shown by microarray.

**Conclusions:**

From these data, it appears plausible that during isolation of the retina, trace amounts of lens tissue may end up in the studied retinal samples. This would explain the high level of variability in transcript levels of genes, the strong correlation of relative levels between samples, and the link with lens-specific function of the "altered" genes. Changes in crystallin gene expression in other models of retinal degeneration have been reported and a careful examination of the transcript level of other lens-specific genes is essential to rule out a possible confounding effect of lens-material transfer.

## Introduction

The gradual and selective loss of retinal ganglion cells via apoptosis underlies the progression of glaucoma. A variety of causal factors leading to ganglion cell apoptosis has been put forward, but the relative contribution of these factors to the initiation and progression of glaucoma remains unknown. One of the factors implicated in glaucoma as well as in diabetic retinopathy and central retinal artery occlusion, is hypoxia/ischemia [1-4]. The preferential loss of retinal ganglion cells in the glaucomatous retina corresponds to the pattern of neuronal degeneration through apoptosis after experimental ischemia [5,6]. We have initiated a series of experiments in order to study the alterations in gene expression patterns after 60 min of ischemia and after ischemic preconditioning (IPC). The latter refers to the effect that a short period of ischemia, 5 min, does not lead to cell loss but instead provides a transient protection against the degenerative effects of a subsequent full ischemic insult [7,8].

The results of these studies will be published elsewhere [9]. In the present report we focus on the profound changes observed in a set of genes including members of the crystallin gene family and other genes associated with the lens. At first alterations in the expression of crystallin genes seemed to be in line with reports on altered crystallin transcript levels resulting from chronic elevation of intraocular eye pressure [10,11], light injury [12], mechanical injury [13], and genetic retinal degeneration [14]. However, subsequent evaluation of our microarray findings by quantitative PCR assays performed on individual samples revealed an exceptional large inter-individual variation in the expression level of this set of genes, in both control and experimental groups.

The focus of this report was to investigate the possibility that a transfer of tissue from the lens to the retinal sample may occur and corrupt the expression data for genes that are abundantly expressed in the lens compared to the retina.

## Methods

### Animals and anesthetics

Animal handling and experimental procedures were reviewed and approved by the ethical committee for animal care and use of the Royal Netherlands Academy for Sciences, acting in accordance with the European Community Council directive of 24 November 1986 (86/609/EEC) and the ARVO statement for the use of animals in Ophthalmic and Vision Research. Transient retinal ischemia was induced by means of raising the pressure in the anterior chamber of the rat eye via an inserted needle as described in detail previously [15-18]. For ischemic preconditioning (IPC) 5 min of ischemia was applied and retinas were isolated 1, 3, 6, 12, 24, 48 h, and 7 days later (n=5-6, each condition). For ischemia/reperfusion (I/R), 60 min of ischemia was applied and the retinas were studied after the following reperfusion times: 1, 2, 6, and 12 h (n=5/condition). To study the changes in gene expression after 60 min of ischemia in preconditioned animals (IPC-I/R), animals were first subjected to 5 min ischemia followed, after an interval of 24 h, by 60 min of ischemia. Retinas were studied after the following reperfusion times: 1, 2, 6, and 12 h (n=6-7 each time point). Animals were killed by an overdose of sodium-pentobarbital (0.8 ml; 60 mg/ml) intraperitoneally. Sham-operations were performed by inserting a needle into the anterior chamber without elevating the pressure.

### RNA isolation

The eyes were enucleated and washed in cold phosphate buffered saline. A circumferential cut was made along the ora serrata. The cornea, ciliary body, lens and adhering vitreous were carefully removed from the eyecup using a pair of brushes. The retina was separated from the sclera, frozen on dry ice, and stored at -80 °C until use. Frozen retina was thawed in Trizol (Invitrogen) and homogenized. Total RNA was isolated following the manufacturer's instructions. The total RNA yield was 8-10 μg/retina and quality checks showed sharp ribosomal RNA bands with RNA Integrity Numbers (RIN) >8.7 (Agilent Technologies 2100 Bioanalyzer).

Four eye lenses were isolated, placed in Trizol and shaken for 5-10 min. The total RNA sample obtained was used for microarray probe generation and for qPCR assays.

### Microarrays and analysis of microarray data

Of each animal, 1 μg of isolated total RNA was used to make pooled samples for each of the groups and one for all contralateral control retinas (n=36). From these pooled samples amino allyl-UTP labeled aRNA was prepared (Amino Allyl MessageAmp aRNA kit, Ambion) and coupled to Cy3 or Cy5 monoreactive dyes (Amersham). For details see Kamphuis et al. [9].

For microarray hybridization, a common reference design was used. aRNA of the control-group (30 μg) was labeled with Cy3. An aliquot of 1 μg of this sample served as the identical common reference sample present on each array and was hybridized together with 1 μg of Cy5-labeled aRNA from one of the experimental groups. Hybridization and washing was carried out according to the protocols described by Agilent. For details see: "Agilent" 60-mer oligo microarray processing protocol v2.1 (SSPE/SureHyb)® at Agilent. After normalization, the resulting dye ratio represents the transcript ratio between the two hybridized samples.

### Microarrays

Oligonucleotide arrays were obtained from Agilent (22K catalog rat array, 60-mer oligonucleotides, product number G4130A; detailed information on all oligonucleotide probes can be found at Agilent or Geo).

### Data analysis

The array images were acquired using an Agilent microarray scanner set at 5 μm resolution and were processed with Feature Extraction software v8.1 (Agilent) using default settings for all parameters. The Feature Extraction output shows the sequence identification and description of all chip oligonucleotide probes, the signal strength of the treatment (Cy5) and control (Cy3) channels, the relationship between the two channels in terms of log_10_ ratio, the estimated log ratio error and associated p-value. Images and data files were uploaded to the Rosetta Biosoftware Resolver® system, v5.0. Detailed information on the Rosetta Resolver system can be found at Rosettabiosoftware, and in several published papers [19-21].

### Web tools

Gene annotation of the genes on the array was updated using web-based annotation tools DAVID and Source. Of the 20,280 features representing expressed sequences 18,690 were linked to a UniGene Cluster ID (92%). Gene Ontology (GO) analysis was performed with *GO*stat (GOstat) [22]. Searches for regulatory elements in the crystallin genes were conducted with the Genomatix Suite (Genomatix) [23], and with Match 1.0 (Generegulation).

### Real-time quantitative polymerase chain reaction

For qPCR, 2 μg of total RNA from each individual retina was DNaseI treated, reverse transcribed into cDNA with 100 U Superscript III Reverse Transcriptase (Invitrogen) and 50 ng random hexamer primers. The resulting cDNA sample served as a template for SYBR green RT-qPCR analysis (ABI 7300 real-time system). The amplification efficiencies were close to 100% for all primer combinations. The resulting C_t_ values were converted to absolute amounts of transcript present in the sample (E*^-C^*^t^) in arbitrary units, multiplied by 10^10^ for presentation purposes [24].

To correct for differences in cDNA load between the different samples, we normalized the target PCR to a set of four reference PCRs. For this purpose, qPCR data for a selection of candidate reference genes on experimental and control samples was used as input for a geNorm analysis [24,25]. *Rho* (rhodopsin), *Hprt* (hypoxanthine phosphoribosyl transferase), *Pde6b* (phosphodiesterase 6B), and *Prkca* (protein kinase C alpha) were identified as most stable and were used for normalization. All presented transcript levels, including crystallin levels, are normalized against the transcript levels of these reference genes.

## Results

### GO-analysis

Three different conditions of retinal ischemia were studied: ischemic preconditioning (IPC), ischemia-reperfusion (I/R), and IPC followed by ischemia-reperfusion 24 h later (IPC-I/R). Applying a feature extraction assigned p value <0.001 cutoff on the microarray-derived changes showed that several members of the crystallin-gene family were highly regulated under all three conditions with 2- to 10 fold changes. Gene ontology (GO) analysis [22], revealed a statistically significant overrepresentation of the GO-term Structural constituent of eye lens (GO id: 0005212) for most of the experimental conditions (Table 1).

**Table 1:** Gene ontology results for the changes in gene expression.

**Group**	**N**	**Genes**	**C/T**	**P**
IPC 1 hour	57	Cryaam Cryab, Cryba1, Crybb1, Crybb2, Lim2	6/20	6.8×10^-12^
IPC 3 hour	59	Bfsp1, Cryaa, Cryab, Cryba1, Cryba4, Crybb1, Crybb2, Crybb3, Crygd, Lim2	10/20	1.0×10^-22^
IPC 6 hour	53	Bfsp1, Cryaa, Cryab, Cryba1, Cryba4, Crybb1, Crybb2, Crybb3, Crygd, Lim2	10/20	2.5×10^-23^
IPC 12 hour	15	Cryba1	1/20	n.s.
IPC 24 hour	25	Bfsp1, Cryaa, Cryba1, Crybb1, Crygd	5/20	8.1×10^-12^
IPC 48 hour	31	Cryba1, Crygd	2/20	1.9×10^-4^
IPC 7 days	10	-	0/20	n.s.
I/R 1 hour	250	Cryaa, Cryba1, Cryba4, Crybb1, Crybb2, Crygd	6/20	1.1×10^-8^
I/R 2 hour	209	Bfsp1, Cryaa, Cryab, Cryba1, Cryba4, Crybb1, Crybb2, Crybb3, Crygd, Lim2	10/20	3.4×10^-15^
I/R 6 hour	783	Cryaa, Cryab, Cryba1, Cryba4, Crybb1, Crybb2, Crybb3, Crygd, Lim2	9/20	2.9×10^-10^
I/R 12 hour	1218	Cryab, Crygd	2/20	n.s.
IPC-I/R 1 hour	413	Bfsp1, Cryaa, Cryab, Cryba1, Cryba4, Crybb1, Crybb2, Crybb3, Crygd, Lim2	10/20	3.3×10^-15^
IPC-I/R 2 hour	593	Bfsp1, Cryaa, Cryab, Cryba1, Cryba4, Crybb1, Crybb2, Crygd, Lim2	9/20	1.3×10^-11^
IPC-I/R 6 hour	887	Cryaa, Cryab, Cryba1, Cryba4, Crybb1, Crybb2, Crybb3, Crygd, Lim2	9/20	4.9×10^-10^
IPC-I/R 12 hour	1925	Bfsp1, Cryaa, Cryab, Cryba1, Cryba4, Crybb1, Crybb2, Lim2	8/20	2.1×10^-6^

Changes in gene expression (p<0.0001 and >1.7) fold associated to the GO term GO:0005212 (Structural constituent of eye lens) after IPC, I/R and IPC-I/R. The rat Genome database (RGD) was used for GO gene association. The microarray design covers 11 of the 20 genes assigned to this GO-term. n represents total number of features showing a >1.7 fold change, C/T; number of signatures genes assigned to GO-term/total number of genes associated to GO: 0005212 in RGD, p-value; Fisher's exact test, n.s represents not significant.

### Cluster analysis

**Figure 1:**
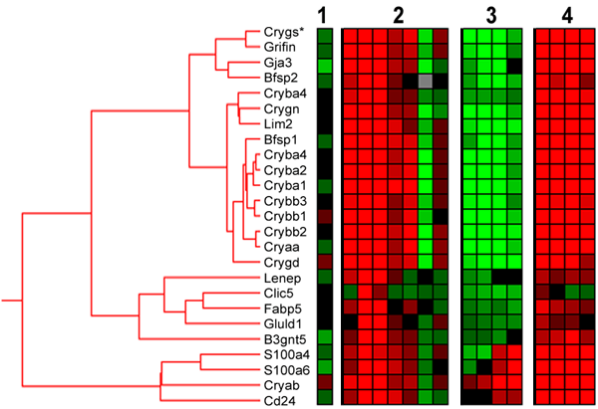
Hierarchical clustering of changes in lens-specific transcripts. Hierarchical clustering of the combined data set: (1) self-self array; (2) IPC; (3) I/R, and (4) IPC-I/R. A total of 25 features, representing 23 different genes, were included in this cluster with a basic node at R^2^>0.75. Color scale ranges between >2 fold upregulation (red) and >2 fold downregulation (green) compared to corresponding control groups. Gray indicates missing data points. For more details on the genes in the cluster see Table 2. Note on "*Crygs*": The annotation for the microarray feature listed as *Crygs* (XM_573311) may not be correct. A qPCR primer set was designed unique for the rat *Crygs* with RefSeq (NM_001012016), but the expression levels assessed did not show a correlation with the other cluster members and the lens/retina ratio was 0.09, showing a greater abundance in the retina. These qPCR results for *Crygs* are not in line with our hypothesis. Blast analysis performed for the 60-mer *Crygs* probe sequence on the array confirmed a match with XM_573311 in the 3'-UTR and also with *Crygs* of mouse and human but *not* with RefSeq NM_001012016. It may that the probe in fact recognizes other members of the *Cryg* family. qPCR assays were developed for rat *Cryga*, *Crygb*, and *Crygc*. The comparison of the lens and retinal transcript levels resulted in a lens-to-retina ratio of 73, 3273, and 106138, respectively. Expression levels showed a close correlation with the other cluster members. The conclusion is that the *Crygs* probe on the array is most likely not specific for *Crygs* but interrogates *Cryga*, *Crygb*, and/or *Crygc*.

Clustering was carried out on the changes observed in the three different experimental conditions separately and on the combined total data set. Various clustering algorithms consistently identified a set of 25 features, representing 23 different genes, with a parallel response to the different ischemic conditions (Figure 1). This set included 10 different crystallin genes (*Cryaa, Cryab, Cryba1/ba3, Cryba2, Cryba4, Crybb1, Crybb2, Crybb3, Crygd, Crygn*) and six genes with a known lens-association: *Bfsp1* (Beaded filament structural protein 1, also known as filensin), *Bfsp2* (Beaded filament structural protein 2, also known as *phakinin* or *CP49*), *Gluld1* (Glutamate-ammonia ligase domain containing 1/lens glutamine synthase-like (*lengsin*)), *Grifin* (Galectin-related inter-fiber protein), *Lenep* (lens epithelial protein), and *Lim2* (Lens intrinsic membrane protein 2). The transcript levels of these genes were transiently upregulated after IPC by 4- to 7 fold at 1, 3 and 6 h. Following I/R in naive animals, transcript levels were *decreased* 3- to 10 fold. In contrast, in preconditioned animals, ischemia resulted in a persistent *increase* of 2- to 6 fold compared to controls. The crystallin features *Crym*, *Cryac*, and *Cryz* were not associated with the cluster.

### qPCR

**Figure 2:**
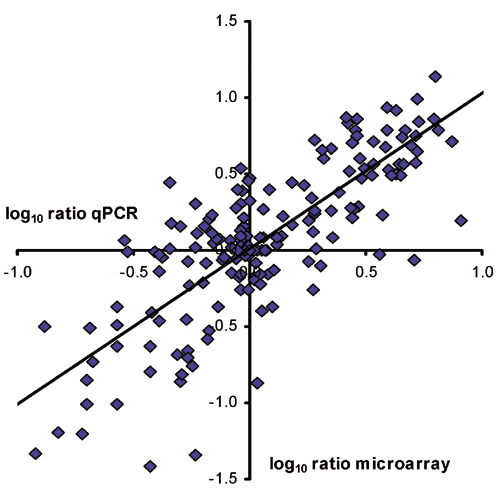
Gene expression changes determined by microarrays versus Qpcr. Results for the 14 different crystallin genes on the array are presented for all groups. The trend line: y=1.02x+0.01; R^2^=0.61.

The transcript levels of the 14 crystallin genes were investigated by qPCR assays on all individual RNA-samples that contributed to the sample pools. Linear regression analysis of the fold change as measured on microarray against the average change derived from the qPCR results showed a significant correlation (R^2^=0.61; Figure 2). qPCR data were also obtained for a different group of 16 other transcripts, including the reference genes (*Gapd, Hprt, Pde6b, Prkca*), genes known to give an early response to ischemia (*c-fos, c-jun*) and genes known to show a more delayed response (*Hmox1, Gfap, Vim, Gria1, Gria2, Pvalb, Thy1, nestin, VIP, Thy1*). The correlation coefficient R^2^ for this group was at 0.73 similar to the set of crystallin genes. However, the two groups of genes differed in the level of variance among the individual samples. For the crystallins the variance was 120% (SD/mean * 100%), while the variance for the second group of genes was around 40% comparable to our earlier findings [18,24]. The difference in variance was consistent in groups of non-treated animals, sham-operated animals, and in all three experimental groups. As a consequence of this high variance, most changes depicted in Figure 1 were not found to be statistical significant whereas ischemia-induced alterations of *c-fos, c-jun, Hmox1, Gfap, Vim, Gria1, Gria2, Pvalb, Thy1, nestin, VIP,* and *Thy1* were all highly significant [18,24]. The only significant changes found in the group of crystallin genes were the increased *Cryab* and *Cryac* expression levels at 2, 6, and 12 h after I/R and after IPC+I/R (range 4- to 8 fold; p<0.001; Student's t-test).

**Figure 3:**
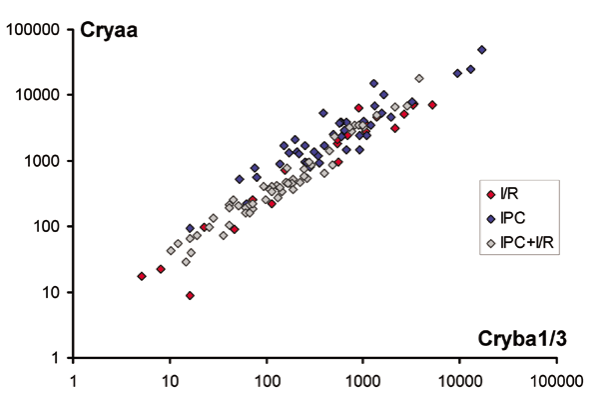
Correlation plot for *Cryaa* and *Cryba1/3* transcript levels in retinal cDNA samples as an example for the high correlation in transcript levels of the cluster genes. All samples of the combined data set were included (R^2^=0.93). Note the overlap between the three experimental groups and the wide range of transcript levels found. Transcript levels were normalized against the levels of the four reference genes.

In contrast to the high inter-individual variance, the transcript levels between different cluster genes showed strong correlations. An example showing the close correlation between *Cryaa* and *Cryba1/3* is presented in Figure 3. This implies that the expression levels of the different cluster genes are linked and that transcript levels have a consistent expression profile, differing between individual samples only by a scaling factor. The transcript levels of crystallins not assigned to the cluster (*Crym*, *Cryac*, and *Cryz*) did not show this correlation.

These findings may indicate a co-regulation for the expression of this set of genes. However, genes in the cluster are located on different chromosomes thus ruling out a tandem arrangement of these genes under the control of a single promotor region. Expression could also be united by a common transcription factor simultaneously acting on all genes. *Pax6*, one of the transcription factors inferred in the regulation of crystallin gene expression may be a candidate for such a role [26], however changes in *Pax6* transcript levels were not in line with the pattern presented in Figure 1. Other transcription factors implicated in the regulation of crystallin gene expression were either not altered (*c-Maf, Sox-2, Six3, Hsf2, Ap1, Foxd3, Foxj2, Hsf, Oct1, v-Maf*), or were not represented on the array (*MafB, Sox1, Ror, Prox1*). Searches for transcription binding sites in the crystallin genes in rat and mouse genome yielded potential regulation sites for large families of transcription factors ETSF, HOXF, EVE1, GATA, and CREB. Attempts to find a common framework of multiple transcription factor binding sites did not lead to results.

Another explanation for the correlated transcript levels could be the unintentional transfer of lens tissue during the isolation of the retina. Genes that are abundantly expressed in lens and with a low expression level in the retina would display highly correlated levels. To test this hypothesis, 2 μg RNA samples from the lens and retina (n=4 each) were analyzed by qPCR. The results are presented in Table 2. Of the set of 24 genes identified by clustering, 17 were studied by qPCR and 16 were found to much more abundant in the lens with an average lens/retina ratio of 2,624; ranging from 215 (*S100a4*) up to 12,700 (*Crybb2*). In addition *Crygb*, a crystallin gene not represented on the array, had a lens/retina ratio of 3273, and the *Crygb* levels were found to be correlated to the transcript levels found for the cluster genes. Moreover, the crystallins *Cryac, Cryz, Crym* not assigned to the cluster, had ratios of 0.32, 0.09, and 0.006, respectively, showing a higher expression in the retina compared to expression in the lens.

**Table 2:** Symbol and accession number information on genes of the cluster given in figure 1.

**Unigene symbol**	**Unigene Cluster**	**GenBank**	**Locus**	**qPCR Lens**	**qPCR Retina**	**Ratio L/R**	**Description**
Grifin	Rn.26894	NM_057187	12q11	47127	15	3190	Galectin-related inter-fiber protein
Gja3	Rn.10345	TC541283	15p12	n.d.	n.d.	n.d.	Connexin 46.
Bfsp2	Rn.40868	XM_238549	8q32	n.d.	n.d.	n.d.	Similar to beaded filament structural protein 2, phakinin, similar to cytoskeletal protein 49
Crygn	Rn.23610	XM_216055	4q11	167	0.08	2037	Crystallin, gamma N (predicted)
Lim2	Rn.9688	NM_053771	1q22	4779	2.6	1809	Lens intrinsic membrane protein 2 (19 kDa)
Bfsp1	Rn.9785	XM_342529	3q41	3785	1.4	2690	Lens fiber cell beaded-filament structural protein
Cryba4	Rn.10802	NM_031689	-	60340	12	5104	Crystallin, beta A4
Cryba2	Rn.93025	NM_173140	9q33	102729	43	2396	Crystallin, beta A2
Cryba1/3	Rn.20326	XM_340846	10q24	205888	83	2490	Crystallin, beta A1 (predicted)
Crybb3	Rn.19693	NM_031690	-	25081	13	1941	Crystallin, beta B3
Crybb1	Rn.10602	NM_012936	-	18431	15	1264	Crystallin, beta B1
Crybb2	Rn.10350	NM_012937	-	16948	1.3	12700	Crystallin, beta B2
Cryaa	Rn.127769	NM_012534	-	764877	520	1472	Crytallin, alpha A
Crygd	Rn.64655	NM_033095	9q32	60590	23	2634	Crystallin, gamma D
Lenep	Rn.10187	NM_053614	2q24	n.d.	n.d.	n.d.	Lens epithelial protein
Clic5	Rn.1838	NM_023025	9q12	n.d.	n.d.	n.d.	Chloride intracellular channel 5
Fabp5	Rn.98269	NM_145878	-	n.d.	n.d.	n.d.	Fatty acid binding protein 5, epidermal
Gluld1	Rn.38685	NM_181383	9q21	n.d.	n.d.	n.d.	Glutamate-ammonia ligase (glutamine synthase) domain containing 1 (lengsin)
B3gnt5	Rn.144639	AW916093	11q23	n.d.	n.d.	n.d.	UDP-GlcNac:betaGal beta-1,3-N-acetylglucosaminyltransferase 5
S100a4	Rn.504	NM_012618	2q34	2205	10	215	S100 calcium-binding protein A4
S100a6	Rn.3233	NM_053485	2q34	2717	2.0	1355	S100 calcium binding protein A6 (calcyclin)
Cryab	Rn.98208	NM_012935	8q23	209182	256	818	Crystallin, alpha B
Cd24	Rn.6007	NM_012752	20q13	6854	8.4	816	CD24 antigen

Transcript levels in lens and retina are determined by qPCR and are presented in arbitrary units. n.d. represents not determined by qPCR.

We investigated the possibility that transfer of vitreous could be implicated because it is difficult to remove all overlying vitreous from the retina during isolation. The amount of RNA in all of the vitreous obtained from a single rat eye is low (50-125 ng; n=4). qPCR assays were performed for *Gapd*, *Hprt*, *28S* and *Bfsp1*, *Lim2*, *Grifin*, *Crym*, *Cryaa*, *Crybb3*. The profile of transcript levels was comparable to that of the retinal samples. However, *Rho*, *Pde6b* and *Thy1* transcripts were detected in the vitreous samples, indicating a contamination with retinal tissue. The normalized transcript levels of *Rho*, *Pde6b* and *Thy1* were comparable to those found in retinal samples.

### Microarray of lens versus retina

**Figure 4:**
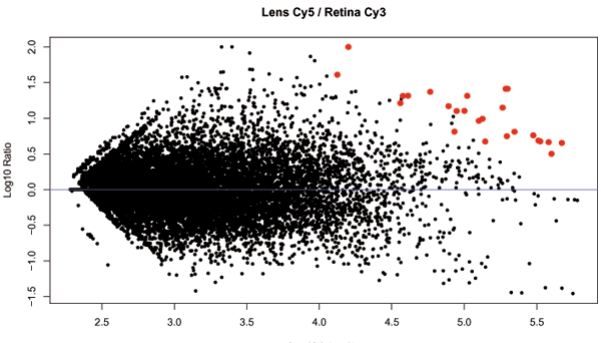
MA plot. Vertical axis represents log10 Cy5 (lens)/Cy3 (retina). Horizontal axis represents log10 average intensity of both channels. Red data points show the position of the 25 features found in the cluster.

In order to verify whether the set of 25 genes of the cluster are all genes with a high lens to retina expression ratio, we performed a microarray hybridization of the lens samples against retinal control samples. All 25 features found in the cluster had high expression levels in the lens compared to the retina; the PMT-settings of the scanner had to be reduced to 2% to prevent saturation of these features. The resulting MA-plot is shown in Figure 4.

## Discussion

We conducted a series of microarray experiments on rat retina to study alterations in the gene expression profiles after transient ischemia in order to gain insight in molecular pathways that lead to neurodegeneration and neuroprotection [7,8,27-30]. The results of these studies will be published elsewhere; here we focus on the marked changes detected in a cluster of 23 genes, including crystallins and other lens-specific genes *Bfsp1, -2, Gluld1, Grifin, Lenep*, and *Lim2*. These genes showed parallel changes after IPC, I/R, and after IPC-I/R. These findings are based on microarray data of pooled samples. The pooling strategy was prompted by the high cost of the arrays. It must be emphasized that pooling is less favorable from a statistical point of view, in particular for genes with higher variability in expression level, making validation by qPCR on individual samples a necessity [31]. Nevertheless, previously reported alterations in gene expression were confirmed by the microarray results [18,24], and changes detected by microarrays were validated by qPCR [9]. The qPCR validation of the cluster genes revealed a higher variability of inter-individual transcript levels compared to a set of other genes [18,24,32,33], and consequently, the ischemia-related changes in expression were not statistically significant. Only the increase of *Cryab* and *Cryac* transcript levels was confirmed. This may be of physiological relevance since Cryab confers a cytoprotective effect on various cell types and has been implicated in several neurological diseases like multiple sclerosis, Alzheimer, Parkinson disease [34,35], and myocardial ischemia [34]. qPCR results also showed that the transcript levels between the different cluster genes were tightly coupled over the wide range of absolute levels. A common framework of transcription factor binding at the gene level could not be identified, which makes a biological regulatory mechanism to explain this observation unlikely.

We therefore explored the possibility that a transfer of lens material into the isolated retinal tissue underlies the coupling in transcript levels of these genes. In accordance with this hypothesis we found that: (i) the transcript levels of the cluster genes were between 215-times and 12,700-times more abundant in RNA isolated from the total lens as compared RNA from the retina, (ii) when comparing equal amounts of lens and retinal mRNA by microarray, the cluster genes had the highest lens/retina ratio. The same cluster genes were not abundantly expressed in samples of the vitreous. In conclusion, a transfer of lens tissue into the isolated retinal sample offers a plausible explanation of our observations.

The most likely moment of transfer to take place is during isolation of the retina. The rat lens is relatively large and the lens may be damaged when the circumferential cut along the ora serrata is made. The cornea, ciliary body, lens, and vitreous were removed using a pair of brushes. These brushes were cleaned in buffer and then used to pick up the retina from the eyecup and transfer it to a vial for storage. Following this method, small amounts of lens tissue may contaminate the isolated retinal sample. We tested an alternative method for isolation of the retina on a separate series of 59 eyes; opening the eye with a cut through the cornea and taking out the lens, vitreous and retina simultaneously, to avoid damaging the lens, followed by dissection of the retina in buffer. However, this method also resulted in crystallin transcript levels with a variation coefficient of 133%, indicating that transfer may be difficult to avoid completely. RNA isolation by laser dissection from retinal cryosections may be effective in preventing contamination but this method is labor-intensive and may compromise the quality of RNA (unpublished observations N.Y.).

The lens contains lens epithelium, stem cells, and lens fiber cells in the transition zone undergoing terminal differentiation into secondary lens fibers. The superficial lens fiber cells are nucleated and have the standard complement of intracellular membrane compartments. Crystallins α, β, and γ are ubiquitously expressed in the more superficial cortical fiber cells positioned at the equatorial region [36-38], together with *Bfsp1*, *Bfsp2* [39] and *Gja3* [40]. *Aqp0* also known as *Lim1*, another major lens protein, was not represented on our array [37]. Recently, Hawse et al. [41] compared the gene expression profile of human lens epithelial cells and lens cortical fiber cells by microarray analysis, and found an enhanced expression of several crystallins (*Cryba1, -ba2, -ba4, -bb1, -bb3, -gA, -gB, -gC, and -gD*), *Bfsp1, Bfsp2, Lim2, Lenep, Clic5*, and *Cd24* in fiber cells compared to epithelial cells. These results support our hypothesis that lens fiber cells from the superficial layers of the lens is most likely source of inadvertently transferred material.

Our data show that the transcript levels of the genes of the cluster are low in the retina. In contrast, expression of the *Crym*, *Cryz* and *Cryac*, which were not in the cluster, are more abundant in the retina. Localization studies on crystallins have confirmed strong Crym-immunostaining in photoreceptors but not in the lens [42]. In mouse retina, expression of all of the 20 studied crystallin genes was detected by qPCR but similar to our findings with highly variable levels [32]. Moreover, immunoblotting showed a high variation for Cryaa, Cryab, Crybb2, and Cryg (b, c, d) protein levels of more than 5- to 10 fold compared to tubulin levels.

To gain insight into retinal transcript levels in samples not corrupted by lens material, we analyzed microarray data collected from human photoreceptor RNA isolated by laser dissection from cryosections (unpublished observations NY). Detectable hybridization was found for all cluster members but the signal strength was lower than 4% of the maximum intensity on the array, indicating low transcript levels. The hybridization signals for *Gryab*, *Cryz*, *Crym*, and *Crygs* were strongest, which is in good agreement with our findings in the rat. In conclusion, there is ample evidence for retinal gene expression of crystallins but transcript levels are low. For that reason, due to high transcript levels found in the lens, contamination of retinal tissue with traces of lens tissue will greatly bias these transcript levels.

An intriguing question is whether our findings have any relevance for the changes in retinal crystallin gene expression that have been reported for several experimental models involving retinal injury or degeneration. As can be concluded from our data, a proper statistical analysis is essential to evaluate the observed changes. For instance, light-induced retinal damage was claimed to lead to a 2- to 3 fold increase in spot intensity of all identified crystallins in 2D-gels, however statistical information was not given [12]. A microarray study on retinal ischemia reported an upregulation of *Cryaa*, *Cryab*, *Cryba1/3, Crybb2, Crygc*, and *Grifin* transcript levels after 12 h of reperfusion, but no qPCR validation was presented [43]. Chronic elevation of intra-ocular pressure in rats resulted in decreased levels of *Cryaa, Crybb2*, and *Cryab* at 8 days after episcleral vein injection but the effect disappeared after 35 days and was not confirmed by qPCR [10]. Two studies showed evidence for changes of crystallin expression in the retina and were validated by qPCR [11,13]. Local mechanical injury of the rat retina was found to be accompanied by an enhanced expression for all crystallins on the microarray and of *Lim2*, *Bfsp1*, and *Grifin* [13]. The upregulation for *Cryab* and *Crygd* was confirmed by qPCR and the injury was accompanied by an increased immunoreactivity for crystallin-β,-α, and γ near the scar [13]. A microarray study on the glaucomatous DBA/2J mouse identified a loss of *Cryaa, Cryba1, Cryba2, Cryba4, Crybb1, Crybb3, Crygb, Cryd, Cryn, Cd24, Grifin, Lim2*, and *Mip* (*Lim1*), of which *Cryba4* and *Mip* were confirmed by qPCR [11].

In conclusion, studies on changes in the retinal transcriptome have often reported alterations of crystallins and several other lens-associated genes. Our observations show that the data on these genes have the risk of being corrupted by transfer of lens material into the isolated retinal tissue. Avoiding pooling strategies in microarray designs, a careful statistical evaluation of individual samples, and the use of comparative lens cDNA samples as controls, is essential to draw any conclusions on gene expression changes in the retina of genes with an abundant expression in the lens relative to the retina.
